# Histone deacetylase inhibitors mitigate antipsychotic risperidone-induced motor side effects in aged mice and in a mouse model of Alzheimer’s disease

**DOI:** 10.3389/fpsyt.2022.1020831

**Published:** 2023-01-06

**Authors:** Guadalupe Rodriguez, Daniel W. Fisher, Bryan McClarty, Janitza Montalvo-Ortiz, Qiaoling Cui, C. Savio Chan, Hongxin Dong

**Affiliations:** ^1^Department of Psychiatry and Behavioral Sciences, Northwestern University Feinberg School of Medicine, Chicago, IL, United States; ^2^Department of Psychiatry and Behavioral Sciences, University of Washington School of Medicine, Seattle, WA, United States; ^3^Department of Neuroscience, Northwestern University Feinberg School of Medicine, Chicago, IL, United States

**Keywords:** antipsychotics, HDAC inhibitors, risperidone, neuropsychiatric symptoms, epigenetic, motor side effects, aging, Alzheimer’s disease

## Abstract

Antipsychotic drugs are still widely prescribed to control various severe neuropsychiatric symptoms in the elderly and dementia patients although they are off-label use in the United States. However, clinical practice shows greater side effects and lower efficacy of antipsychotics for this vulnerable population and the mechanisms surrounding this aged-related sensitivity are not well understood. Our previous studies have shown that aging-induced epigenetic alterations may be involved in the increasing severity of typical antipsychotic haloperidol induced side effects in aged mice. Still, it is unknown if similar epigenetic mechanisms extend to atypical antipsychotics, which are most often prescribed to dementia patients combined with severe neuropsychiatric symptoms. In this study, we report that atypical antipsychotic risperidone also causes increased motor side effect behaviors in aged mice and 5xFAD mice. Histone deacetylase (HDAC) inhibitor Valproic Acid and Entinostat can mitigate the risperidone induced motor side effects. We further showed besides D2R, reduced expression of 5-HT2A, one of the primary atypical antipsychotic targets in the striatum of aged mice that are also mitigated by HDAC inhibitors. Finally, we demonstrate that specific histone acetylation mark H3K27 is hypoacetylated at the *5htr2a* and *Drd2* promoters in aged mice and can be reversed with HDAC inhibitors. Our work here establishes evidence for a mechanism where aging reduces expression of 5-HT2A and D2R, the key atypical antipsychotic drug targets through epigenetic alteration. HDAC inhibitors can restore 5-HT2A and D2R expression in aged mice and decrease the motor side effects in aged and 5xFAD mice.

## Introduction

Though antipsychotics are primarily selected for treatment of psychoses, these pharmaceuticals are often being applied to control the severe neuropsychiatric symptoms in dementia patients with an off-label use in the United States (1–3). Alzheimer’s Disease (AD), the most common type of dementia, encompasses 60–80% of all dementias (4, 5). Over 90% of neuropsychiatric symptoms occur in AD patients, making these symptoms an integral part of this disease (6). As the US population is progressing toward a record number of individuals reaching advanced age, the number of AD cases are likely to rise precipitously, reflecting potential increased use of antipsychotics to treat these symptoms. Despite the frequent off-label use of antipsychotics in the elderly, these drugs have been shown to have decreasing efficacy and increased side-effects specifically in elderly populations, most notably with the emergence of higher risk of developing extrapyramidal side effects, stroke, and death (7, 8). Recent concern regarding the higher mortality rate observed in elderly patients prescribed antipsychotic drugs has prompted the FDA to require a “black box” warning on marketing materials (9, 10). The mechanism of these increased side-effects and lower efficacy is likely multifactorial, with pharmacokinetic and pharmacodynamic changes indicated (11, 12). Of these mechanisms, epigenetic influences at gene promoters for antipsychotic targets seems to be a key driver of the narrowing therapeutic window with age (13–15). In particular, our group has demonstrated that reduced dopamine receptor 2 (D2R; gene name *Drd2*) expression leads to increased extrapyramidal side effects with the typical antipsychotic haloperidol (14, 15). This reduced expression of D2R is driven by hypoacetylation of histones at the gene promoter for *Drd2* in the striatum and reversal of the hypoacetylation with histone deacetylase (HDAC) inhibitors leads to decreased extrapyramidal side effects, specifically in aged but not in young mice (14, 16). Combined treatment of antipsychotic with HDAC inhibitor to widen the therapeutic window represents a novel strategy to increase functionality and reduce side-effects with these drugs in the elderly population (11, 15).

Despite this initial promising work with typical antipsychotics, the vast majority of elderly patients are treated with atypical antipsychotics, such as risperidone, olanzapine, and quetiapine (17). Although the receptor profiles for every antipsychotic are complex, typical antipsychotics favor blockade of D2R while atypical antipsychotics display greater antagonism of the serotonin receptor 2A (5-HT2A, gene name *5htr2a*) (18, 19), much less antagonism of the D2R. While in general atypical antipsychotics display fewer extrapyramidal side effects and greater safety profiles overall in the elderly (20, 21), the motor side effects of these drugs are still a significant concern in clinical practice (2). In this study, we determined the severity of risperidone induced motor side effects with dose response in aged mice and 5xFAD mice, a common mouse model of AD (22). Additionally, we investigated whether adjunct HDAC inhibitor treatment with risperidone could mitigate such side effects. Risperidone was selected as it is the only federally approved drug in Europe to treat neuropsychiatric symptoms in AD (23) and has the higher rate of extrapyramidal side effects compared to other atypical antipsychotics (24, 25). We further investigated how 5-HT2A and D2R expression changes during aging, and after HDAC inhibitor administration. Finally, we characterized histone acetylation mark H3K27ac at the *5htr2a* and *Drd2* promoter to determine whether these epigenetic changes could be implicated in the differences in atypical antipsychotic efficacy and tolerability in the elderly.

## Materials and methods

### Animals

Young (2–3 months old, *n* = 6–8 per group) and aged (20–24 months, *n* = 5–6 per group) C57BL/6 J mice and 5xFAD mice and wildtype (WT) littermates at 11 months of age (*n* = 8–19) of both sexes from JAX laboratory [B6SJL-Tg(APPSwFlLon,PSEN1*M146L*L286V)6799Vas/Mmjax] were used for this study (RRID:MMRRC_034840-JAX). Animals were group housed on a 12-hour light/dark cycle and given food and water *ad libitum*. All procedures were performed according to NIH guidelines for the treatment of animal subjects and the Current Guide for the Care and Use of Laboratory Animals (2011, eighth edition) under a protocol approved by the Northwestern University Animal Care and Use Committee.

### Drugs

All drugs were purchased from Sigma (St. Louis, MO, USA). Risperidone (RIS; 0.01, 0.5, and 0.1 mg/kg) was prepared in 0.9% saline with pH adjusted to 5–6, titrated with 0.1 M NaOH. Valproic Acid (VPA, 400 mg/kg) was dissolved in 0.9% saline with a pH of 7.3 and Entinostat (MS-275, 10 mg/kg) was dissolved in DMSO and 0.9% saline with pH adjusted to 5–6 (13, 14). Vehicle (Veh) was used as control. All treatments were administered by intra-peritoneal injection (IP) at a constant volume of 10 ul/g of body weight once a day for 14 consecutive days. VPA or MS-275 was injected 30 min before RIS administration.

### Behavioral tests

Prior to behavioral procedures, animals were acclimated for at least 30 min to a soundproof behavioral testing room. Assays were performed during the light portion of the 24-hour light/dark cycle. Arenas and behavioral testing equipment were cleaned with 70% ethanol between each trial.

#### Catalepsy

Catalepsy is widely used for testing extrapyramidal side effects induced by antipsychotics in preclinical studies (26–29) including our group (14). We performed the catalepsy assay as described in our previous publications (14, 16). Briefly, a 1-cm diameter plastic rod was suspended 3.5 cm above a laboratory bench. Thirty minutes after all drug administration, the front paws of the mouse were placed on the rod while the hind paws rested on the bench. The duration of a cataleptic episode was defined and recorded as the latency to remove the forepaws from the rod during a 300-s (5 min) trial.

#### Open field

The open field test was applied for evaluation of the locomotor activity (14). Briefly, each mouse was placed in a Plexiglass box (25 cm × 25 cm × 25 cm) that was evenly illuminated in a soundproof testing room. Any-Maze Automated tracking system was used to track open field locomotor activity. Locomotion was measured as distance traveled in centimeters during a 10-minute period.

#### Rotarod

The TSE Rotarod System (Bad, Homburg, Germany) was used to assess motor coordination after drug administration. Mice were placed on an accelerating rod (4–40 rpm during the first 5 min) for 10 min, and the latency to fall from the rod was recorded. A total of three trials were conducted with a 10-min inter-trial interval. The average latency to fall from the rod across the three trials was calculated and used for comparison (14, 30).

#### Western blot

After behavioral test, the brain tissues were collected through a cardiac perfusion with 0.1 M PBS solution for 1 min, to wash-out the blood from blood vessels in the mouse brain. The brains were then removed and quickly dissected under ice with a dissecting scope. Tissues were frozen at −80°C until ready for processing for molecular analysis. For immunoblotting measuring the 5-HT2A and D2R expression levels, striatum tissue samples were collected and homogenized in an ice-cold RIPA buffer (Sigma, R0278) and centrifuged at 12,000 rpm for 20 min at 4°C. Protein quantification was measured using Pierce™ BCA Protein Assay Kit (ThermoFisher Scientific, 23225) according to protocol provided by the manufacturer. Polyvinylidene difluoride (PVDF) membranes (Biorad, 1620177) were used to transfer protein samples. Blots were blocked for 1-hour with 5% non-fat dry milk (Biorad, 1706404) and incubated using 1:1,000 5HT2A (Boster Biological, PA1373), 1:500 D2R (Millipore, AB5084P), or 1:1000 B-actin (Santa Cruz, sc-47778) overnight at 4°C. Blots were then incubated for 2 h at room temperature using secondary 1:3,000 goat anti-rabbit (Biorad, 170-6515) or 1:3,000 goat anti-mouse (Biorad, 172-1011). Blotted protein bands were detected using ChemiDoc imaging system (Biorad) and quantified using Image J Software. The levels of 5HT2A and D2R protein expression were normalized to β-actin (14, 16).

#### Chromatin immunoprecipitation (ChIP) assay

For measuring histone acetylation marks, the Magna ChIP G Tissue Kit (Millipore, 17-20000) assay was used and performed according to the kit protocol. Fragmented chromatin lysate was precipitated with 2–3 ul of antibody for H3K27ac (Abcam, ab4729). The DNA-histone complex was incubated with salmon sperm DNA/protein A-agarose beads overnight to estimate non-specific binding. The DNA-histone complex was eluted from the beads and dissociated at 65° for 4 h in a water bath under high-salt conditions. Proteins were digested using proteinase-K treatment, and the associated DNA was precipitated with 100% ethanol and re-suspended in 50 ul of PCR-grade water (14, 16).

#### Quantitative real-time PCR (qRT-PCR)

Levels of interaction between modified histones and the *5htr2a* gene promoter were determined by measuring the amount of histone-associated DNA, isolated *via* chromatin immunoprecipitation (ChIP) and quantified using quantitative real-time PCR (qRT-PCR). We used custom primers for the 5-HT2A promoter (forward: 5′CTGGACCGCTACGTGGCTAT-3, reverse: 5′TATGGTCCACACCGCAATGA-3′), *Drd2* promoter (forward: 5′-CTGGAGCCAAAAGCAGTCTG-3′, reverse: 5′-TCCTTCAGGTTTCCGACGCC-3′), and the b-actin promoter (forward: 5′-GCGTCCACCCGCGAGTACAA-3′, reverse: 5′-TCCATGGCGAACTGGTGGCG-3′) used as a control. Input and immunoprecipitated DNA amplification reactions were run in triplicate in the presence of SYBR Green (Applied Biosystems, 4472908) using an ABI Prism 7,700 thermocycler. Ct values from each sample were obtained using the Sequence Detector 1.1 software. Ct values were normalized to the endogenous gene, b-actin, to obtain a percent input. Fold differences (drug-treated vs. control) were then determined using the △△Ct method (14, 16).

#### Statistical analysis

All statistical analyses were conducted using Graph Prism Pad (San Diego, CA, USA). Data are expressed as mean ± standard error of the mean (S.E.M.). Catalepsy, locomotor activity, and rotarod data were analyzed using two-way analysis of variance (ANOVA) to detect age, treatment, and interaction effects followed by a multiple comparison analysis using Bonferroni’s *post-hoc* method. Significance was set at *p* < 0.05.

## Results

### Risperidone induces greater motor side effects in aged mice with dose response manner

In our study, we found that chronic RIS administration in aged mice displayed more severe motor side effects similar to the typical antipsychotic drug haloperidol measured through the behavioral tests we have applied before (14, 16).

#### Catalepsy

**FIGURE 1:**
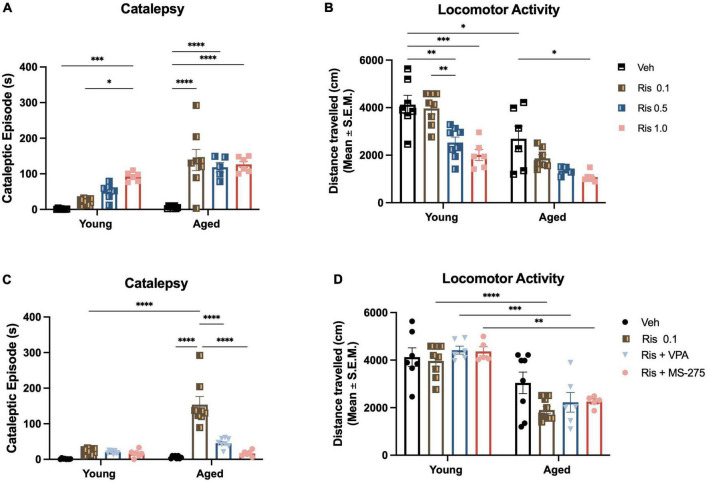
Risperidone induces a dose response of motor side effects in aged mice and histone deacetylase (HDAC) inhibitors could prevent the side effects. **(A)** Motor side effects measured by cataleptic episode in 5 min. Lower dose of RIS at 0.1 mg/kg already showed a significant increase in cataleptic behavior in aged mice but not young mice. However, the higher doses of RIS could cataleptic behavior in both young and aged mice, although more severe in aged mice. **(B)** General locomotor activity measured by open field. Again, the lower dose of RIS at 0.1 mg/kg displayed a decrease in the distance traveled in aged mice but not in young group. However, higher doses of RIS at 0.5 and 1.0 mg/kg induced the decreased in the distance traveled with a dose respond manner. **(C)** Co-treatment of lower dose of RIS (0.1 mg/kg) with VPA (400 mg/kg) or MS-275 (10 mg/kg) reduced the duration of catalepsy in aged mice. **(D)** Lower dose of RIS (0.1 mg/kg) with VPA (400 mg/kg) or MS-275 (10 mg/kg) did mitigate the RIS-induced decrease in locomotor activity in the young but not old mice. Data represent mean ± SEM (*n* = 6–8/group). *p < 0.05, ^**^p < 0.01, ^***^p < 0.001, ^****^p < 0.0001.

Two-way ANOVA showed significant effects of age (F_1,47_ = 32.88, *p* < 0.0001), drug (F_3,47_ = 24.25, *p* < 0.0001), and age and drug interaction (F_3,47_ = 7.06, *p* = 0.0005) on the duration of cataleptic episodes. In aged groups, *post-hoc* analysis revealed significant increases in the duration of cataleptic episodes at all doses selected in RIS as compared to their age-matched control mice (all *p* < 0.0001). There was no difference of cataleptic episodes between RIS treated groups (0.1–1.0 mg/kg) (Figure 1A). In addition, aged mice showed a greater degree of catalepsy than young mice at each RIS dose administered. In young groups, RIS induced cataleptic episodes are increased with dose response displaying a significant increase in the duration of cataleptic episodes at dose of 1.0 mg/kg (*p* = 0.0005). These data are comparable to the results we observed with haloperidol in aged mice (13, 14).

#### Locomotor activity

Two-way ANOVA showed significant effects of age (F_1,44_ = 45.00, *p* < 0.001) and drug (F_3,44_ = 16.64, *p* < 0.001) but no interaction between age and drug (F_3,44_ = 1.55, *p* = 0.2155) on the distance traveled in the open field. In the aged groups, RIS- induced decrease in the distance traveled displayed a dose response with higher doses being more significant (0.5 mg/kg, *p* < 0.05 and 1.0 mg/kg, *p* < 0.01) (Figure 1B). In young groups, the decrease in the distance traveled also displayed a dose response (0.5 mg/kg, *p* < 0.01; 1.0 mg/kg *p* < 0.001). However, there were no significance in the distance traveled between young and aged group.

### HDAC inhibitor mitigate the risperidone induced motor side effects

After behavior tests for dosing response of RIS in young and aged mice, we determined whether HDAC inhibitor could reverse such effect. We selected the dose of RIS at 0.1 mg/kg as this dose is comparable to the dose that use for elderly patients and showed the motor side effects in aged mice but not young mice.

#### Catalepsy

Two-way ANOVA showed significant effects of age (F_1,53_ = 41.11, *p* < 0.0001), drug (F_3,53_ = 36.35, *p* < 0.0001), and age and drug interaction (F_3,53_ = 24.28, *p* < 0.0001) on the duration of cataleptic episodes. Specifically, *post-hoc* analysis demonstrated that either VPA or MS-275 can reduce the duration of catalepsy for the aged mice given a 0.1 mg/kg dose of RIS (both *p* < 0.0001) (Figure 1C). There were no differences detected between VPA or MS-275 on RIS-induced catalepsy duration. Similar to our results for haloperidol, these data demonstrate that atypical antipsychotic-induced catalepsy can be mitigated with adjunct HDAC inhibitor treatment.

#### Locomotor activity

Interestingly, Two-way ANOVA did not show any significant effects of age (F_1,47_ = 69.31, *p* < 0.0001), drug (F_3,47_ = 1.76, *p* = 0.1673), or interaction of drug and age (F_3,47_ = 1.50, *p* = 0.2263) in the distance traveled. *Post-hoc* analysis showed there were no differences in the distance traveled in the Veh treated groups between young and aged groups. RIS administration significantly decreased the distance traveled in the aged mice as compared to young mice (*p* < 0.001) (Figure 1D). However, adjunct treatment RIS with HDAC inhibitor VPA and MS275 did not mitigate the RIS-induced decrease in locomotor activity in aged mice (Figure 1D). Similar to our previous work with haloperidol, the atypical antipsychotic RIS reduced locomotion but this was not reversed with either HDAC inhibitor.

### Risperidone induces greater motor side effects in 5xFAD mice that can be reversed with HDAC inhibitors

Antipsychotics are often prescribed to dementia patients to control some severe behavioral and psychological symptoms, such as agitation, aggression, and psychosis Yurusa (31). In this study, we evaluated whether the typical antipsychotic drug RIS could induce more severe side effects on 5xFAD mice at 12 months of age, when the memory deficits and neuropathology manifest, and whether HDAC inhibitors could reverse such side effects.

#### Catalepsy

**FIGURE 2:**
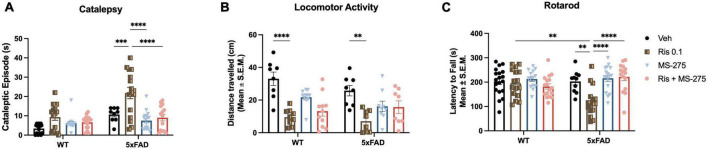
Risperidone induces motor side effects in 5xFAD mice and histone deacetylase (HDAC) inhibitor could prevent the side effects. **(A)** Lower dose of RIS (0.1 mg/kg) induced the cataleptic episode in 5xFAD but not wild type (WT) mice at 12 months of age. Co-treatment of RIS with MS-275 significantly reduced cataleptic episodes of 5xFAD mice. **(B)** RIS induced reduced general locomotor function in both WT and 5xFAD mice measured by the distance traveled in the open field. However, co-treatment of RIS with MS-275 did not improve general locomotor activity. **(C)** RIS also effected on motor coordination in 5xFAD mice but not WT controls by decreasing the latency to fall measured the rotarod. Co-treatment of RIS with MS-275 significantly improved motor coordination. Data represent mean ± SEM (*n* = 8–19/group). ^**^p < 0.01, ^***^p < 0.001, ^****^p < 0.0001.

Two-way ANOVA revealed significant effects of genotype (F_3,111_ = 25.90, *p* < 0.0001) and drug (F_3,111_ = 14.21, *p* < 0.0001), and genotype and drug interaction (F_3,111_ = 56.13, *p* < 0.0001) on cataleptic episodes (Figure 2A). *Post-hoc* analysis revealed 5xFAD mice but not WT mice administered RIS displayed severe cataleptic behavior compared to 5xFAD Veh treated mice, and WT RIS treated mice (both *p* < 0.0010). However, 5xFAD mice co-treated with MS-275 showed a significant decrease in cataleptic behavior, when compared to 5xFAD mice treated with RIS alone (*p* < 0.0001). These results suggest selective vulnerability of atypical antipsychotic drug induced cataleptic behavior in 5xFAD mice compared to WT controls, and co-treatment with MS-275 can reduce RIS-induced catalepsy in 5xFAD mice.

#### Locomotor activity

Two-way ANOVA only revealed a significant effect of drug (F_3,64_ = 17.36, *p* < 0.0001) but not genotype and drug and genotype interaction in the total distance traveled (Figure 2B). *Post-hoc* analysis showed a significant decreased in the total distance traveled in WT RIS treated mice compared to WT Veh treated mice (*p* < 0.0001). Similarly, 5xFAD RIS treated mice showed a significant decrease in the total distance traveled compared to 5xFAD Veh treated mice (*p* = 0.0014). No significant difference was found between Veh and RIS treated groups between WT and 5xFAD mice. Co-administration of RIS and MS-275 did not significantly improve general locomotor activity. These results suggest that general locomotor activity declines with chronic RIS administration, regardless of genotype, however, chronic administration of MS-275 is not sufficient to improve general locomotion in both RIS treated WT and 5xFAD mice.

#### Rotarod

Two-way ANOVA revealed a significant effect of drug but not genotype (F_3,116_ = 7.27, *p* < 0.0001), and a genotype and drug interaction (F_3,116_ = 6.90, *p* < 0.0001) on the latency to fall from the rod (Figure 2C). *Post-hoc* analysis showed a significant decline of motor coordination in 5xFAD mice treated with RIS compared to Veh (*p* < 0.01), however, no significant difference in motor coordination was found in WT RIS treated mice compared to WT Veh treated mice. Additionally, RIS treated 5xFAD mice showed a significant decrease in motor coordination when compared to RIS treated WT mice (*p* < 0.01). Furthermore, co-treatment of RIS and MS-275 showed a significant increase of motor coordination in 5xFAD mice compared to RIS treated 5xFAD mice (*p* < 0.0001). These results suggest atypical antipsychotic drug induced motor coordination dysfunction specifically in 5xFAD mice and co-treatment with MS-275 can improve motor coordination in RIS treated 5xFAD mice.

### Aging decreased 5-HT2A expression in the striatum in aged mice and HDAC inhibitors reversed this expression

**FIGURE 3:**
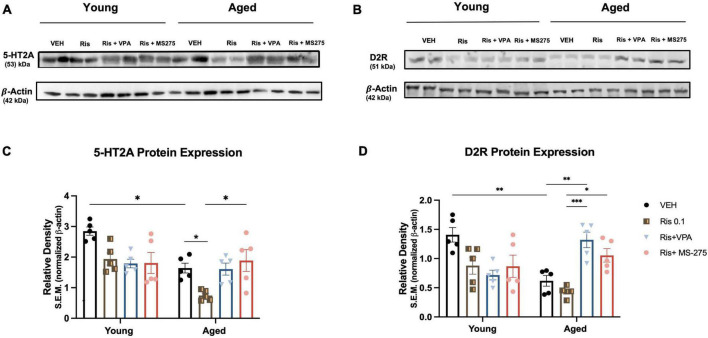
Effects of histone deacetylase (HDAC) inhibitors on 5-HT2A and D2R protein expression in the striatum of young and aged mice. **(A, C)** 5-HT2A expression was significantly decreased in the striatum in aged mice but not young mice, RIS further induced decreased expression in of 5-HT2A expression in aged mice. Co-treatment of RIS with VPA and MS-275 could restore 5-HT2A expression in aged mice. **(B, D)** D2R expression was decreased in the striatum in aged mice as compared to young mice administration of both VPA and MS-275 with RIS significantly increased D2R expression in the striatum as compared to Veh and RIS treated aged mice. Data represent mean ± SEM (*n* = 5). *p < 0.05, ^**^p < 0.01, ^***^p < 0.001.

Atypical antipsychotics primarily target 5-HT2A, therefore, we examined whether this receptor is decreased in aged mice and whether RIS administration differentially affects expression of this receptor with aging. We analyzed 5-HT2A expression in the striatum *via* western blot in young and aged mice treated with Veh, RIS, and HDAC inhibitors VPA or MS-275. Two-way ANOVAs showed significant effects of age (F_1,32_ = 15.90, *p* = 0.0004), drug (F_3,32_ = 5.68, *p* = 0.0031) and age and drug interaction (F_1,32_ = 4.59, *p* = 0.0088) on 5HT2A protein levels in the striatum. *Post-hoc* testing indicated a significantly decreased 5-HT2A expression in aged mice compared to young mice for Veh treated groups (*p* = 0.0112). Moreover, RIS induced a significant decrease in 5-HT2A protein levels in the striatum in aged mice (*p* < 0.05) but not young mice although young mice displayed a tendency of decreased 5-HT2A expression but not significant (Figures 3A, C). However, in aged but not young mice, significant increases in 5-HT2A protein levels were found in co-treated RIS and MS-275 (*p* = 0.0124) mice and a trend of increased 5-HT2A protein levels in co-treated RIS and VPA mice as compared to those treated with RIS alone. Overall, the atypical antipsychotic target 5-HT2A is reduced with aging, and chronic treatment of RIS further reduces 5-HT2A expression, which can be restored with HDAC inhibitor in old but not young mice in the striatum as compared to RIS group (Figures 3A, C).

### Aging alters D2R expression in the striatum in aged mice and HDAC inhibitors reversed this expression

In addition to 5-HT2A, atypical antipsychotics also target D2R, therefore, in this study we also measured D2R expression in the striatum. Two-way ANOVA revealed significant effect of drug (F_3,32_ = 3.93, *p* = 0.0017) but not age (F_1,32_ = 1.62, *p* = 0.2119), and an age and drug interaction (F_3,32_ = 12.79, *p* < 0.0001) in D2R expression in the striatum (Figures 3B, D). *Post-hoc* analysis showed a significant decrease in D2R expression in aged mice as compared to young mice in Veh treated groups (*p* = 0.0018). Administration of RIS showed a trend of decreased D2R expression, however, it was not significant in both ages. Administration of either VPA or MS-275 with RIS significantly increased D2R expression in the striatum of aged mice as compared to Veh (*p* = 0.001) and RIS treated (*p* < 0.0001) aged groups. No significant differences were found in young mice between treatment groups.

### HDAC inhibitor increases histone acetylation at the *5htr2a* gene promoter in young and aged mice

**FIGURE 4:**
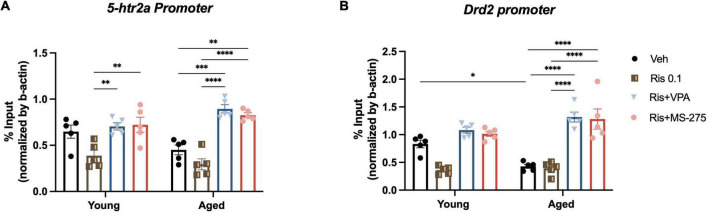
Effects of histone deacetylase (HDAC) inhibitors on histone acetylation at the *5htr2a* and *Drd2* gene promoters in the striatum in young and aged mice. **(A)** No significant differences of H3K27ac binding at the *5htr2a* promoter between young and aged group was found, but a significant increase in H3K27ac binding at the *5ht2a* promoter in the striatum was found in aged and young mice co-treated with RIS and VPA or MS-275 compared to those treated with RIS alone. **(B)** H3K27ac binding at the *Drd2* promoter was significantly decreased in aged mice as compared to young mice in Veh treated group. However, H3K27ac binding at the *Drd2* promoter in the striatum was observed to be significantly increased in aged mice co-treated with RIS and HDAC inhibitors VPA or MS-275 when compared to those treated with Veh alone. Data are presented as mean ± SEM, (*n* = 5). *p < 0.05, ^**^p < 0.01, ^***^p < 0.001, ^****^p < 0.0001.

We previously found that histone hypoacetylation at the *Drd2* gene promoter in the striatum led to the reduced protein expression and lower tolerability of typical antipsychotic drug haloperidol (14, 16). To determine whether similar changes of histone modification can be observed on the *5htr2a* gene with aging, we used ChIP to measure histone acetylation marker H3K27ac, at the *5htr2a* gene promoter in the striatum of young and aged mice treated with combinations of Veh, RIS, and HDAC inhibitors. Two-way ANOVA showed significant effect of drug (F_3,32_ = 28.52, *p* < 0.0001) and an interaction of drug and age (F_3,32_ = 4.86, *p* = 0.0066), but not age (F_1,32_ = 0.00015, *p* = 0.9902), in the binding of H3K27ac to the *5htr2a* promoter. *Post-hoc* analysis found no significant differences of H3K27ac level binding to the *5htr2a* promoter in Veh treated groups between young and aged mice, but RIS did show a trend of decreasing H3K27ac levels in both groups. Interestingly, a significant increase in H3K27ac level binding to the *5htr2a* promoter was found in young and aged mice co-treated with RIS and VPA (*p* < 0.001) and RIS and MS-275 (*p* < 0.001) compared to those treated with Veh or RIS alone (Figure 4A).

### HDAC inhibitor decreases RIS induced-motor side effect through restoring histone acetylation at the *Drd2* gene promoter in aged mice

Given *Drd2* plays a significant role in antipsychotic induced side effects, we then investigated if the severe motor side effects observed in RIS treated mice are due to hypoacetylation at *Drd2* promoter resulting in the decreased D2R expression and function. ChIP was used to measure histone acetylation marker H3K27ac levels at the *Drd2* gene promoter in the striatum in aged and young mice treated with Veh, RIS, and HDAC inhibitors. Two-way ANOVA showed significant effect of drug (F_3,32_ = 46.13, *p* < 0.0001), and a drug and age interaction (F_3,32_ = 6.84, *p* = 0.0011), but not age (F_1,32_ = 0.30, *p* = 0.5907) in the binding of H3K27ac to the *Drd2* gene promoter in the striatum. *Post-hoc* analysis found a significant decrease in H3K27ac level binding at the *Drd2* promoter in aged mice as compared to young mice treated with Veh. Moreover, significant increase in H3K27ac level binding at the *Drd2* promoter was found in aged but not young mice co-treated with RIS and VPA (*p* < 0.0001) and RIS and MS-275 (*p* < 0.0001) compared to those treated with Veh (Figure 4B).

## Discussion

In this study we aimed to investigate whether atypical antipsychotics could induce more severe motor side effects in aged mice and 5xFAD mice, an animal model of AD. We also explored whether the changes of histone modification at antipsychotic target genes play a significant role in response to such side effects in aging. We found that atypical antipsychotic RIS leads to greater motor side-effects in aged mice as well as 5xFAD mice, and that these side-effects can be reversed with adjunct HDAC inhibitor VPA, or MS-275. RIS induced age-related motor side effects are similar to that of typical antipsychotic drug haloperidol (14, 16), confirming the increased sensitivity of atypical antipsychotic drugs in aged mice. We also demonstrated that similar epigenetic regulation at the *Drd2* and *5htr2a* gene promoters is hypoacetylated at a key residue H3K27ac and can be reversed with adjunct HDAC inhibitor in aged mice.

Due to the higher risk of extrapyramidal side effects, such as tardive dyskinesia and death, typical antipsychotics have been replaced by atypical antipsychotics for the treatment of psychiatric disorders as well as to control severe psychiatric symptoms of AD in the elderly (32–34). In the US, there are currently no FDA-approved drugs to treat behavioral psychiatric symptoms in AD and antipsychotics have a black box warning due to the increase in mortality in elderly patients treated with these drugs (9, 10). Despite this, RIS, one of the most common antipsychotics prescribed to elderly patients, is approved to treat psychiatric symptoms in dementia in Europe, Canada, New Zealand, and Australia (23). In general, atypical antipsychotics, including RIS, have decreased the extrapyramidal side effects as compared to the typical antipsychotic haloperidol (35). However, in elderly patients, the higher risk of motor side effects still exists. For this reason, we chose to study this medication in our models of aging and AD to reveal if an epigenetic mechanism we found in our previous work with typical antipsychotic drug, haloperidol, is similar in our study with atypical antipsychotic drug RIS.

As expected, RIS induced very similar motor side effects to haloperidol in aged mice. Both haloperidol and risperidone caused an increase in catalepsy in aged compared to young mice but do not cause greater age-dependent hypolocomotion in the open field test. In addition, adjunct treatment with a broad HDAC inhibitor, VPA, and a more selective Class 1 HDAC inhibitor, MS-275, reversed the age-exaggerated increase in catalepsy without altering the cataleptic responses of young mice to RIS. These results suggest that transcriptional restriction of antipsychotic drug targets by histone hypoacetylation due to aging can be reversed with HDAC inhibitors to increase the therapeutic window of these drugs in elderly patients, expanding the pre-clinical evidence for reducing side-effects with this adjunct treatment to atypical antipsychotics. The mechanism of extrapyramidal side effects such as catalepsy are largely tied to the degree of antagonism, receptor occupancy, and receptor kinetics of D2R or imbalance of D2R and 5-HT2A (23, 36–38). Additionally, out of the atypical antipsychotics, RIS displays the highest antagonism at these two receptors, suggesting that the age-related increases in catalepsy seen with typical antipsychotics and RIS depend on D2R and 5-HT2A expression functioning. In fact, evidence suggests that concomitant 5-HT2A antagonism expands the therapeutic window of antipsychotics through indirect or direct cross-talk with D2R (39, 40). Thus, to investigate the age-related 5-HT2A and D2R expression as well as histone modification on the gene promoters will help to better understand the age-related increase in catalepsy by RIS. In our study, we showed significant reduction of both 5-HT2A and D2R expression in the striatum of aged mice as compared to young mice (Veh groups), and 0.1 mg/kg RIS selectively further reduced 5-HT2A expression in aged mice but not in young mice, although young mice showed a trend to reduce the expression, it was not significant. These results support that decreased 5-HT2A and D2R expression at the base line during aging, which may contribute to the increased sensitivity to antipsychotic drug side effects. Additionally, these results suggest that young and aged mice in response to antipsychotic drugs are differently expressed at the cellular level. After HDAC inhibitor treatment, MS-275 increased both 5-HT2A and D2R expression and VPA selectively increased D2R expression in the striatum in aged mice but not young mice, suggesting HDAC inhibitors could restore antipsychotic drug target receptor expression in aged mice. Our results determined the effects of age and RIS treatment on 5-HT2A and D2R expression and histone acetylation at the *5htr2a* and *Drd2* promoters. Similar to our findings with D2R, aging also leads to a reduction in 5-HT2A expression that is associated with histone hypoacetylation at the *5htr2a* promoter, with alterations in individual acetylated histone marks depending on age, RIS treatment status, and HDAC inhibitor. The same pattern of age-related hypoacetylation and transcriptional repression we found on the D2R in this study and previous report (14, 16), which suggests that epigenetic changes during aging may broadly affect numerous antipsychotic drug targets.

Our current and previous work make a compelling case for adjunct HDAC inhibitors to improve the therapeutic efficacy for antipsychotics in both aged mice as well as the mouse model of AD. However, we have mentioned that 12-month-old 5xFAD mice, which genetically overexpressed APP/PS1, two mutation caused familiar AD but are not naturally aged. Currently, we did not find significant changes D2R and 5-HT2A expression in our AD model, but a trend of decreased D2R and 5-HT2A in the striatum (data not shown) and these results need further confirmation. Nevertheless, we propose that AD neuropathogenesis could influence drug targeted genes that may cause motor side effects. Indeed, antipsychotics are among some of the least selective medications in terms of receptor antagonism, and it is still unknown how aging and/or AD pathogenesis influence D2R, 5-HT2A and other important target receptors, including histaminergic, adrenergic, muscarinic, sigma, and serotonergic and dopaminergic receptors in other brain areas (41). In addition, although the imbalance of D2R and 5-HT2A are one potential reason, 5-HT2A-related side effects have yet to be described conclusively in the literature (42, 43). Future work will be needed to determine if aging-related epigenetic influences at histaminergic, adrenergic, and muscarinic gene promoters lead to greater metabolic and cardiovascular side-effects that may occur with age and are closely associated with these signaling systems (41). Additionally, adding 18-month-old 5xFAD mice as a comparison will provide important information for age and neuropathology interaction effect on antipsychotic actions.

Regarding acetylation at *5ht2a* and *Drd2* promoters, we found that H3K27 levels were significantly decreased in *Drd2* promoter as compared to young mice but have a trend to decrease of H3K27 levels at *5-htr2a* promoter in both young and aged mice. As expected, we found chronic HDAC inhibitor treatment significantly increased *Drd2 and 5-htr2a* gene promoters in aged mice compared to Veh treated aged mice. Although we found a trend of increased H3K27 levels at both *Drd2 and 5-htr2a* gene promoters in young mice when compared to the Veh treated group, that trend did not reach significance. These results suggest that the changes of H3K27 acetylation at *Drd2 and 5-htr2a* gene promoters differently in the antipsychotic targeted receptor between young and aged mice. Our current study is focused on histone acetylation specifically H3K27, other histone modification marks may also modulate at *5ht2a* and *Drd2* gene promoters during aging. Therefore, in future work we will include more histone acetylation marks to identify age-related changes of histone acetylation contributing to 5-HT2A and D2R expression. Additionally, Histone Acetyltransferases (HATs), another important regulator system of histone acetylation (44) might be driving the hypoacetylation seen at the *Drd2* or *5htr2a* promoters. It is unclear how the individual balance of specific HDACs and HATs leads to aging-or disease related narrowing of the antipsychotic therapeutic window. As HDAC inhibitors are powerful drugs, which itself could potentially induce the side-effects (45), understanding which individual HDACs or HATs involved in the aging-induced hypoacetylation of key histones would greatly improve the potential for adjunct HDAC inhibitors, as more selective HDAC inhibitors may lead to better tolerability of this adjunct treatment approach in elderly and AD patients.

In summary, our current study demonstrated that typical antipsychotic drug RIS could reduce more severe motor side effects in aged mice and 5xFAD mice. This work also expands our previous findings from typical antipsychotics to atypical antipsychotics in terms of the aging-related mechanism of hypoacetylation of key drug targets and subsequent increase in side-effect severity in aged mice. Future work will determine the mechanisms of epigenetic regulation in AD involved in antipsychotics along with expanding the investigation of side-effects beyond motor side effects that may provide greater evidence for the adjunct HDAC inhibitor treatment as a viable option for AD patients treated with antipsychotics to reduce side-effects and increase efficacy of these important medications.

## Data availability statement

The raw data supporting the conclusions of this article will be made available by the authors, without undue reservation.

## Ethics statement

The animal study was reviewed and approved by Institutional Animal Care and Use Committee (IACUC) Office at Northwestern University.

## Author contributions

GR and HD designed the experiments and supervised the project. GR, BM, and JM-O were responsible for mice handling, drug preparations, and IP injections. GR, BM, and QC conducted the experiments and data collection. HD, DF, JM-O, CC, and GR revised the manuscript. All authors contributed to the article and approved the submitted version.

## References

[1] Tampi RR Tampi DJ Rogers K Alagarsamy S. Antipsychotics in the management of behavioral and psychological symptoms of dementia: maximizing gain and minimizing harm. *Neurodegener Dis Manag.* (2020) 10:5–8. 10.2217/nmt-2019-0036 32027552

[2] Yunusa I Rashid N Demos GN Mahadik BS Abler VC Rajagopalan K. Comparative outcomes of commonly used off-label atypical antipsychotics in the treatment of dementia-related psychosis: a network meta-analysis. *Adv Ther.* (2022) 39:1993–2008. 10.1007/s12325-022-02075-8 35247186 PMC9056477

[3] Jenraumjit R Somboon J Chainan S Chuenchom P Wongpakaran N Wongpakaran T. Drug-related problems of antipsychotics in treating delirium among elderly patients: a real-world observational study. *J Clin Pharm Ther.* (2021) 46:1274–80. 10.1111/jcpt.13423 33768628

[4] Breijyeh Z Karaman R. Comprehensive review on Alzheimer’s disease: causes and treatment. *Molecules.* (2020) 25:5789. 10.3390/molecules25245789 33302541 PMC7764106

[5] Knopman DS Amieva H Petersen RC Chételat G Holtzman DM Hyman BT Alzheimer disease. *Nat Rev Dis Primers.* (2021) 7:33. 10.1038/s41572-021-00269-y 33986301 PMC8574196

[6] Aupperle P. Management of aggression, agitation, and psychosis in dementia: focus on atypical antipsychotics. *Am J Alzheimers Dis Other Demen.* (2006) 21:101–8. 10.1177/153331750602100209 16634465 PMC10833293

[7] Volkow ND Gur RC Wang GJ Fowler JS Moberg PJ Ding YS Association between decline in brain dopamine activity with age and cognitive and motor impairment in healthy individuals. *Am J Psychiatry.* (1998) 155:344–9. 9501743 10.1176/ajp.155.3.344

[8] Kaasinen V Vilkman H Hietala J Någren K Helenius H Olsson H Age-related dopamine D2/D3 receptor loss in extrastriatal regions of the human brain. *Neurobiol Aging.* (2000) 21:683–8. 10.1016/S0197-4580(00)00149-4 11016537

[9] Schneider LS Dagerman KS Insel P. Risk of death with atypical antipsychotic drug treatment for dementia: meta-analysis of randomized placebo-controlled trials. *JAMA.* (2005) 294:1934–43. 10.1001/jama.294.15.1934 16234500

[10] Steinberg M Lyketsos CG. Atypical antipsychotic use in patients with dementia: managing safety concerns. *Am J Psychiatry.* (2012) 169:900–6. 10.1176/appi.ajp.2012.12030342 22952071 PMC3516138

[11] McClarty BM Fisher DW Dong H. Epigenetic alterations impact on antipsychotic treatment in elderly patients. *Curr Treat Options Psychiatry.* (2018) 5:17–29. 10.1007/s40501-018-0134-4 29755923 PMC5943049

[12] Sykes DA Moore H Stott L Holliday N Javitch JA Lane JR Extrapyramidal side effects of antipsychotics are linked to their association kinetics at dopamine D2 receptors. *Nat Commun.* (2017) 8:763. 10.1038/s41467-017-00716-z 28970469 PMC5624946

[13] Montalvo-Ortiz JL Keegan J Gallardo C Gerst N Tetsuka K Tucker C HDAC inhibitors restore the capacity of aged mice to respond to haloperidol through modulation of histone acetylation. *Neuropsychopharmacology.* (2014) 39:1469–78. 10.1038/npp.2013.346 24366052 PMC3988551

[14] Montalvo-Ortiz JL Fisher DW Rodríguez G Fang D Csernansky JG Dong H. Histone deacetylase inhibitors reverse age-related increases in side effects of haloperidol in mice. *Psychopharmacology.* (2017) 234:2385–98. 10.1007/s00213-017-4629-2 28421257 PMC5538925

[15] Keszycki RM Fisher DW Dong H. The hyperactivity-impulsivity-irritiability-disinhibition-aggression-agitation domain in Alzheimer’s disease: current management and future directions. *Front Pharmacol.* (2019) 10:1109. 10.3389/fphar.2019.01109 31611794 PMC6777414

[16] McClarty B Rodriguez G Dong H. Dose effects of histone deacetylase inhibitor tacedinaline (CI-994) on antipsychotic haloperidol-induced motor and memory side effects in aged mice. *Front Neurosci.* (2021) 15:674745. 10.3389/fnins.2021.674745 34690667 PMC8526546

[17] Jeste D Savla G Thompson W Vahia I Glorioso D Martin A Association between older age and more successful aging: critical role of resilience and depression. *Am J Psychiatry.* (2013) 170:188–96. 10.1176/appi.ajp.2012.12030386 23223917 PMC3593664

[18] Li P Snyder GL Vanover KE. Dopamine targeting drugs for the treatment of schizophrenia: past, present and future. *Curr Top Med Chem.* (2016) 16:3385–403. 10.2174/1568026616666160608084834 27291902 PMC5112764

[19] Seeman P. Atypical antipsychotics: mechanism of action. *Can J Psychiatry.* (2002) 47:27–38. 10.1177/070674370204700106 11873706

[20] Gareri P Fazio PD Manfredi VG Sarro GD. Use and safety of antipsychotics in behavioral disorders in elderly people with dementia. *J Clin Psychopharmacol.* (2014) 34:109–23. 10.1097/JCP.0b013e3182a6096e 24158020

[21] Yen YC Lung FW Chong MY. Adverse effects of risperidone and haloperidol treatment in schizophrenia. *Prog Neuropsychopharmacol Biol Psychiatry.* (2004) 28:285–90. 10.1016/j.pnpbp.2003.10.006 14751424

[22] Oblak A Lin P Kotredes K Pandey R Garceau D Williams H Comprehensive evaluation of the 5XFAD mouse model for preclinical testing applications: a MODEL-AD study. *Front Aging Neurosci.* (2021) 13:713726. 10.3389/fnagi.2021.713726 34366832 PMC8346252

[23] Yunusa I Alsumali A Garba AE Regestein QR Eguale T. Assessment of reported comparative effectiveness and safety of atypical antipsychotics in the treatment of behavioral and psychological symptoms of dementia: a network meta-analysis. *JAMA Netw Open.* (2019) 2:e190828. 10.1001/jamanetworkopen.2019.0828 30901041 PMC6583313

[24] Weiden PJ. EPS profiles: the atypical antipsychotics are not all the same. *J Psychiatr Pract.* (2007) 13:13–24. 10.1097/00131746-200701000-00003 17242588

[25] Ho MY Mobini S Chiang TJ Bradshaw CM Szabadi E. Theory and method in the quantitative analysis of “impulsive choice” behaviour: implications for psychopharmacology. *Psychopharmacology.* (1999) 146:362–72. 10.1007/PL00005482 10550487

[26] Bardin L Kleven MS Barret-Grévoz C Depoortère R Newman-Tancredi A. Antipsychotic-like vs cataleptogenic actions in mice of novel antipsychotics having D2 antagonist and 5-HT1A agonist properties. *Neuropsychopharmacology.* (2006) 31:1869–79. 10.1038/sj.npp.1300940 16237379

[27] Fink-Jensen A Schmidt LS Dencker D Schülein C Wess J Wörtwein G Antipsychotic-induced catalepsy is attenuated in mice lacking the M4 muscarinic acetylcholine receptor. *Eur J Pharmacol.* (2011) 656:39–44. 10.1016/j.ejphar.2011.01.018 21269601 PMC3896864

[28] Nishchal BS Rai S Prabhu MN Ullal SD Rajeswari S Gopalakrishna HN. Effect of tribulus terrestris on haloperidol-induced catalepsy in mice. *Indian J Pharm Sci.* (2014) 76:564–7. 25593394 PMC4293692

[29] Luciani KR Frie JA Khokhar JY. An open source automated bar test for measuring catalepsy in rats. *eNeuro.* (2020) 7:ENEURO.488–419. 10.1523/ENEURO.0488-19.2020 32198157 PMC7307632

[30] Kirschbaum KM Hiemke C Schmitt U. Rotarod impairment: catalepsy-like screening test for antipsychotic side effects. *Int J Neurosci.* (2009) 119:1509–22. 10.1080/00207450902984002 19922371

[31] Yunusa I El Helou ML. The use of risperidone in behavioral and Psychological symptoms of dementia: a review of pharmacology, clinical evidence, regulatory approvals, and off-label use. *Front Pharmacol.* (2020) 11:596. 10.3389/fphar.2020.00596 32528275 PMC7256877

[32] Gareri P Fazio PD Fazio SD Marigliano N Ibbadu GF Sarro GD. Adverse effects of atypical antipsychotics in the elderly: a review. *Drugs Aging.* (2006) 23:937–56. 10.2165/00002512-200623120-00002 17154659

[33] Gareri P Segura-García C Manfredi VG Bruni A Ciambrone P Cerminara G Use of atypical antipsychotics in the elderly: a clinical review. *Clin Interv Aging.* (2014) 9:1363–73. 10.2147/CIA.S63942 25170260 PMC4144926

[34] Maust DT Kim HM Seyfried LS Chiang C Kavanagh J Schneider LS Antipsychotics, other psychotropics, and the risk of death in patients with dementia: number needed to harm. *JAMA Psychiatry.* (2015) 72:438–45. 10.1001/jamapsychiatry.2014.3018 25786075 PMC4439579

[35] Mühlbauer V Möhler R Dichter MN Zuidema SU Köpke S Luijendijk HJ. Antipsychotics for agitation and psychosis in people with Alzheimer’s disease and vascular dementia. *Cochrane Database Syst Rev.* (2021) 12:CD013304. 10.1002/14651858.CD013304.pub2 34918337 PMC8678509

[36] Wadenberg ML Kapur S Soliman A Jones C Vaccarino F. Dopamine D2 receptor occupancy predicts catalepsy and the suppression of conditioned avoidance response behavior in rats. *Psychopharmacology.* (2000) 150:422–9. 10.1007/s002130000466 10958084

[37] Hauber W Neuscheler P Nagel J Müller CE. Catalepsy induced by a blockade of dopamine D1 or D2 receptors was reversed by a concomitant blockade of adenosine A(2A) receptors in the caudate-putamen of rats. *Eur J Neurosci.* (2001) 14:1287–93. 10.1046/j.0953-816x.2001.01759.x 11703457

[38] Wadenberg ML Soliman A VanderSpek SC Kapur S. Dopamine D(2) receptor occupancy is a common mechanism underlying animal models of antipsychotics and their clinical effects. *Neuropsychopharmacology.* (2001) 25:633–41. 10.1016/S0893-133X(01)00261-5 11682246

[39] Wynchank D Berk M. Efficacy of nefazodone in the treatment of neuroleptic induced extrapyramidal side effects: a double-blind randomised parallel group placebo-controlled trial. *Hum Psychopharmacol.* (2003) 18:271–5. 12766931 10.1002/hup.476

[40] Meltzer HY Huang M. *In vivo* actions of atypical antipsychotic drug on serotonergic and dopaminergic systems. *Prog Brain Res.* (2008) 172:177–97. 10.1016/S0079-6123(08)00909-6 18772033

[41] Kaar SJ Gobjila C Butler E Henderson C Howes OD. Making decisions about antipsychotics: a qualitative study of patient experience and the development of a decision aid. *BMC Psychiatry.* (2019) 19:309. 10.1186/s12888-019-2304-3 31646985 PMC6806500

[42] Kusumi I Boku S Takahashi Y. Psychopharmacology of atypical antipsychotic drugs: from the receptor binding profile to neuroprotection and neurogenesis. *Psychiatry Clin Neurosci.* (2015) 69:243–58. 10.1111/pcn.12242 25296946

[43] Meltzer HY Matsubara S Lee JC. The ratios of serotonin2 and dopamine2 affinities differentiate atypical and typical antipsychotic drugs. *Psychopharmacol Bull.* (1989) 25:390–2. 2576319

[44] Carrozza MJ Utley RT Workman JL Côt JÉ. The diverse functions of histone acetyltransferase complexes. *Trends Genet.* (2003) 19:321–9. 10.1016/S0168-9525(03)00115-X 12801725

[45] Subramanian S Bates SE Wright JJ Espinoza-Delgado I Piekarz RL. Clinical toxicities of histone deacetylase inhibitors. *Pharmaceuticals.* (2010) 3:2751–67. 10.3390/ph3092751 27713375 PMC4034096

